# Doxycycline, an Inhibitor of Mitochondrial Biogenesis, Effectively Reduces Cancer Stem Cells (CSCs) in Early Breast Cancer Patients: A Clinical Pilot Study

**DOI:** 10.3389/fonc.2018.00452

**Published:** 2018-10-12

**Authors:** Cristian Scatena, Manuela Roncella, Antonello Di Paolo, Paolo Aretini, Michele Menicagli, Giovanni Fanelli, Carolina Marini, Chiara Maria Mazzanti, Matteo Ghilli, Federica Sotgia, Michael P. Lisanti, Antonio Giuseppe Naccarato

**Affiliations:** ^1^Department of Translational Research and New Technologies in Medicine and Surgery, University of Pisa, Pisa, Italy; ^2^Breast Surgery Unit, Azienda Ospedaliero-Universitaria Pisana, Pisa, Italy; ^3^Department of Clinical and Experimental Medicine, University of Pisa, Pisa, Italy; ^4^Fondazione Pisana per la Scienza, Pisa, Italy; ^5^Department of Laboratory Medicine, Azienda Ospedaliero-Universitaria Pisana, Pisa, Italy; ^6^Division of Breast Radiology, Azienda Ospedaliero-Universitaria Pisana, Pisa, Italy; ^7^Translational Medicine, University of Salford, Greater Manchester, Manchester, United Kingdom

**Keywords:** doxycycline, mitochondria, cancer stem cells, translational study, mitochondrial biogenesis

## Abstract

**Background and objectives:** Cancer stem cells (CSCs) have been implicated in tumor initiation, recurrence, metastatic spread and poor survival in multiple tumor types, breast cancers included. CSCs selectively overexpress key mitochondrial-related proteins and inhibition of mitochondrial function may represent a new potential approach for the eradication of CSCs. Because mitochondria evolved from bacteria, many classes of FDA-approved antibiotics, including doxycycline, actually target mitochondria. Our clinical pilot study aimed to determine whether short-term pre-operative treatment with oral doxycycline results in reduction of CSCs in early breast cancer patients.

**Methods:** Doxycycline was administered orally for 14 days before surgery for a daily dose of 200 mg. Immuno-histochemical analysis of formalin-fixed paraffin-embedded (FFPE) samples from 15 patients, of which 9 were treated with doxycycline and 6 were controls (no treatment), was performed with known biomarkers of “stemness” (CD44, ALDH1), mitochondria (TOMM20), cell proliferation (Ki67, p27), apoptosis (cleaved caspase-3), and neo-angiogenesis (CD31). For each patient, the analysis was performed both on pre-operative specimens (core-biopsies) and surgical specimens. Changes from baseline to post-treatment were assessed with MedCalc 12 (unpaired *t-*test) and ANOVA.

**Results:** Post-doxycycline tumor samples demonstrated a statistically significant decrease in the stemness marker CD44 (*p*-value < 0.005), when compared to pre-doxycycline tumor samples. More specifically, CD44 levels were reduced between 17.65 and 66.67%, in 8 out of 9 patients treated with doxycycline. In contrast, only one patient showed a rise in CD44, by 15%. Overall, this represents a positive response rate of nearly 90%. Similar results were also obtained with ALDH1, another marker of stemness. In contrast, markers of mitochondria, proliferation, apoptosis, and neo-angiogenesis, were all similar between the two groups.

**Conclusions:** Quantitative decreases in CD44 and ALDH1 expression are consistent with pre-clinical experiments and suggest that doxycycline can selectively eradicate CSCs in breast cancer patients *in vivo*. Future studies (with larger numbers of patients) will be conducted to validate these promising pilot studies.

## Introduction

Tumor-initiating cells (TICs) share many functional characteristics with normal stem cells and are important drivers of tumor initiation and cancer progression (1–7). As such, new therapies for targeting TICs [a.k.a., cancer stem cells (CSCs)] could be used for cancer prevention. Interestingly, circulating tumor cells (CTCs) can also functionally behave as initiators of tumor formation.

Because of their resistance to conventional anti-cancer treatments (i.e., chemo-therapy and radio-therapy), CSCs are also thought to underpin the cellular and molecular basis of tumor recurrence, distant metastasis and ultimately treatment failure, in most cancer types (1–6). Thus, new treatment strategies are urgently needed to help remedy this unmet clinical need (1–4).

One simplistic idea is to identify novel therapeutic targets that are relatively unique to CSCs, which can be then be inhibited with FDA-approved drugs that show few side effects and have excellent safety profiles (1–3). We recently used this promising approach to identify mitochondria in CSCs as a conserved therapeutic target (7). In this context, the antibiotic doxycycline emerged as an excellent candidate for drug repurposing (8, 9). In 1967, Doxycycline was first approved by the FDA, more than 50 years ago. It shows minimal side effects and is currently used world-wide as a broad-spectrum antibiotic, mainly for the treatment of acne and acne rosacea. Doxycycline has excellent pharmaco-kinetics, with very good oral absorption (~100%) and a long serum half-life (18–22 h), at the standard dose of 200 mg per day.

Doxycycline functionally behaves as a non-toxic inhibitor of mitochondrial biogenesis, because of the evolutionarily conserved similarities between bacterial ribosomes and mitochondrial ribosomes (10–12). Therefore, this “manageable side-effect” of doxycycline could be repurposed as a “therapeutic effect,” to target and inhibit mitochondrial biogenesis in CSCs (13, 14).

Previously, doxycycline has been used clinically to target cancer-associated infections, with promising results, leading to a complete pathological response (CPR) or “remission” in patients with MALT lymphoma (15, 16). Interestingly, this CPR did not correlate with the presence of micro-organisms, possibly suggesting that doxycycline might be acting on the tumor cells themselves.

In 2015, the Sotgia/Lisanti laboratory first demonstrated that doxycycline treatment was sufficient to successfully halt the propagation of CSCs *in vitro* (13, 14). For this purpose, we tested 12 different human tumor cell lines, representing eight different cancer types, such as DCIS, breast [ER(+) and ER(-)], lung, ovarian, pancreatic, and prostate carcinomas, as well as glioblastoma (GBM) and melanoma (13). Remarkably, doxycycline inhibited CSC propagation across this entire panel of diverse cell lines (13).

Further mechanistic studies, using luciferase based assays in MCF7 cells (a human breast cancer cell line) revealed that doxycycline treatment effectively inhibits CSC signaling, across multiple pathways, including Wnt, Notch, Hedgehog and STAT1/3-signaling (14). Therefore, doxycycline is an excellent candidate for drug repurposing, in clinical pilot studies aimed at validating its ability to target CSCs in cancer patients. As such, here we evaluated the ability of doxycycline to target CSCs in breast cancer patients *in vivo*, using well-established CSC markers (CD44 and ALDH1) as a read-out.

The ability of doxycycline to target breast CSCs *in vitro* has already been confirmed independently (17, 18) and extended to several other classes of antibiotics and mitochondrial OXPHOS inhibitors (19–24). Consistent with these findings, mitochondrial mass is increased in CSCs (25, 26) and high expression levels of mitochondrial markers directly correlates with poor clinical outcome in ovarian (27) and breast cancer patients (28).

Finally, as early as 2002, it was first reported that doxycycline effectively reduces bone metastasis, by up to ~60–80%, in an *in vivo* pre-clinical murine model of human breast cancer (29). Mechanistically, these findings may be due to the ability of doxycycline to eradicate CSCs, although this hypothesis was not tested at that time.

## Results

### Description of the breast cancer patient population

**Figure 1:**
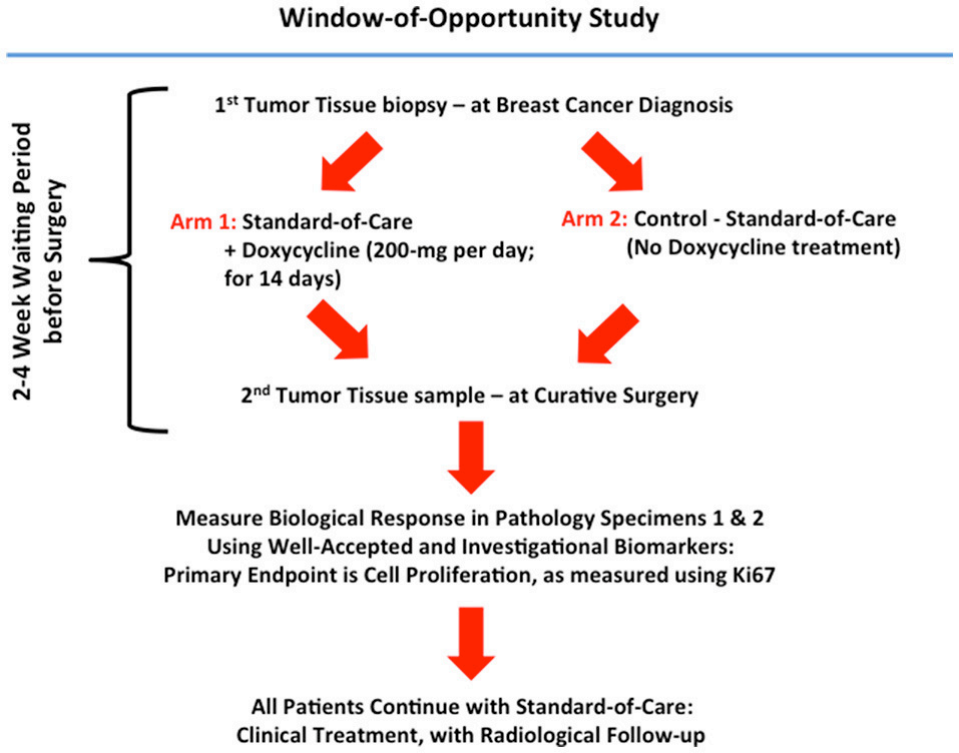
Schematic diagram summarizing the organizational structure of the Doxycycline clinical pilot study. Note that this Phase II “Window-of-Opportunity” format provides an excellent clinical mechanism for evaluating FDA-approved antibiotics, as potential candidates for drug repurposing.

A summary diagram highlighting the organizational structure of this doxycycline “window-of-opportunity” study (Phase II) is shown in Figure 1.

A total of 15 female patients with early breast cancer participated in the current pilot study. Nine patients received doxycycline (200 mg per day) for a 14-day period, while six patients remained untreated. A summary of the clinical characteristics of the patient population are shown in Table 1.

**Table 1:** Clinical characteristics of the patient population.

**Treated patients**	**Age**	**Tumor size (mm)**	**Grade**	**Molecular subtype**
Case 1	42	12	2	Luminal A
Case 2	57	15	3	HER2pos
Case 4	65	23	3	HER2pos
Case 5	52	10	2	Luminal A
Case 7	46	12	2	Luminal A
Case 8	46	27	2	Luminal B
Case 13	50	10	2	Luminal A
Case 14	52	13	1	Luminal A
Case 15	44	30	2	Luminal A
**Untreated patients**
Case 3	71	25	3	Luminal B
Case 6	50	15	2	Luminal A
Case 9	51	12	2	Luminal A
Case 10	48	25	3	Luminal B
Case 11	41	21	2	Luminal A
Case 12	69	16	3	Luminal/HER2pos

Briefly, in the doxycycline treatment group, patient age at diagnosis ranged between 42 and 65 years of age, tumor size was between 10 and 30 mm, and 6 out of 9 patients were grade 2. In addition, 7 out of 9 patients were ER(+), with 6 being of the luminal A sub-type and one of the luminal B sub-type. In addition, two patients were of the HER2(+) sub-type.

In the untreated control group, patient age ranged between 41 and 71 years of age, and tumor size was between 12 to 25 mm; 3 patients were grade 2 and 3 patients were grade 3. All 6 patients were ER(+), with 3 of the luminal A sub-type, 2 of the luminal B sub-type and one showing characteristics of both luminal/HER2(+) sub-types.

Thus, both groups were well-matched for age and clinical characteristics.

### Status of biomarkers in tumor tissue sections, before and after receiving oral doxycycline

We quantitatively assessed the expression of several diverse biomarkers in paraffin-embedded tumor tissue sections. These included markers of “stemness” (CD44, ALDH1), mitochondria (TOMM20), cell proliferation (Ki67, p27), apoptosis (cleaved caspase-3), and neo-angiogenesis (CD31).

**Figure 2:**
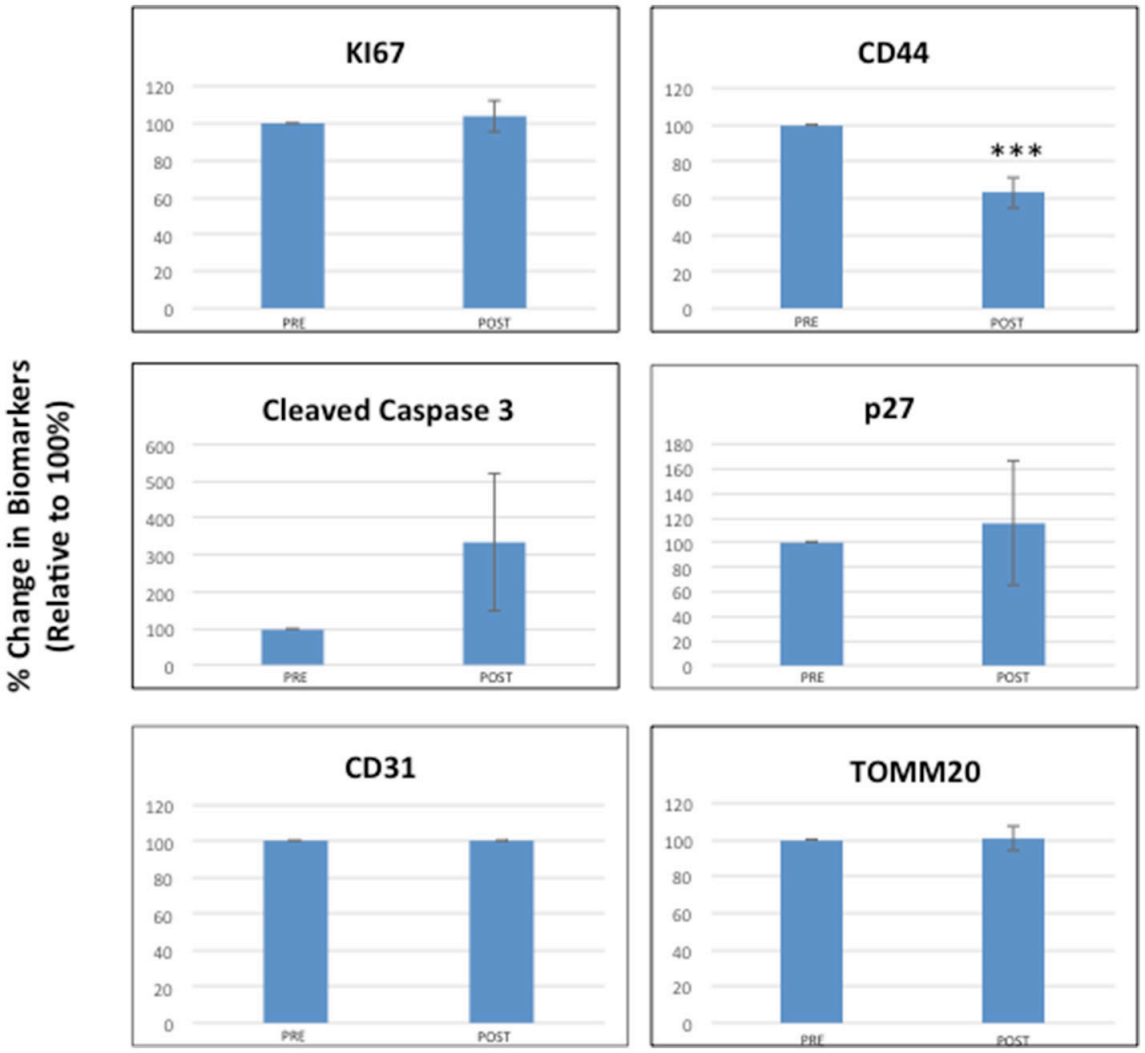
Effects of doxycycline administration on the expression of six different classes of biomarkers in early breast cancer patients (Ki67, Cleaved Caspase-3, CD31, CD44, p27, and TOMM20). Note that only CD44 levels were significantly reduced on average by nearly 40% (***; *p* < 0.005), while the levels of other markers remained unchanged. The results of multi-variate analysis are included as Supplemental Information and show that CD44 remained significant (ANOVA; *p* < 0.0007) and was independent of all the other variables tested [histological grade (1, 2, 3), diameter type (small, large) and molecular subtype] (see Tables S1–S15).

Figure 2 highlights that most of the tumor markers remained unchanged before and after receiving oral doxycycline, with the exception of CD44—a marker of “stemness.” More specifically, CD44 was reduced on average by ~40% (*p* < 0.005), in the patients examined. Note that 4 out of 9 patients showed reductions of 50% or greater in CD44.

The results of multi-variate analysis are included as Supplemental Information and demonstrated that CD44 reductions remained significant (ANOVA; *p* < 0.0007) and were independent of all the other variables tested [including histological grade (1, 2, 3), tumor diameter type (small, large) and molecular subtype] (see Tables S1–S15). In contrast, cleaved caspase-3 levels appeared to be elevated after receiving oral doxycycline; however, this did not reach statistical significance, except in the case of low histological grade (See Table S4).

**Figure 3:**
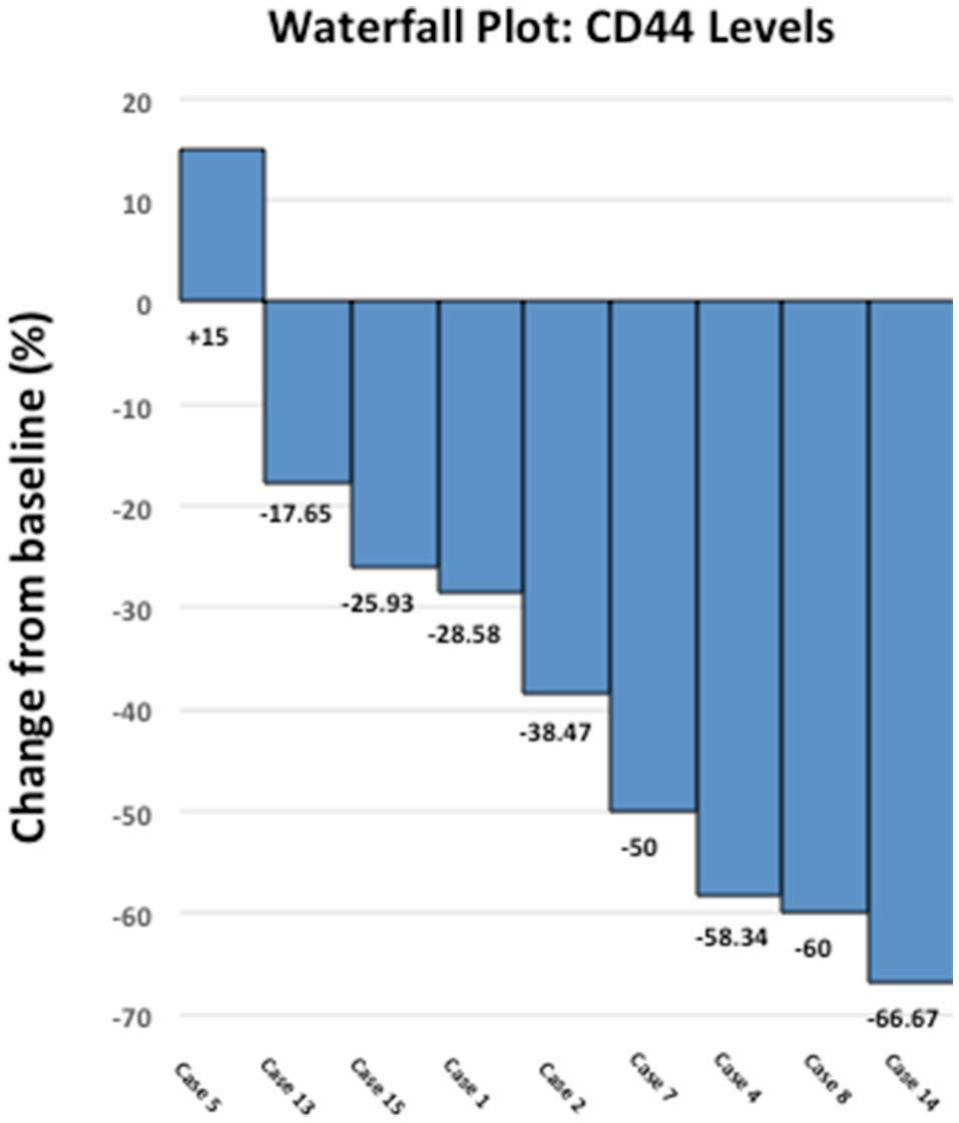
Effect of doxycycline administration on the expression of CD44 in early breast cancer patients: Waterfall plot. Note that CD44 levels were reduced between 17.65 and 66.67%, in 8 out of 9 patients treated. In contrast, only one patient showed a rise in CD44, by 15%. Overall, this represents a positive response rate of nearly 90%. Also, note that 4 out of 9 patients showed reductions of 50% or greater in CD44.

**Figure 4:**
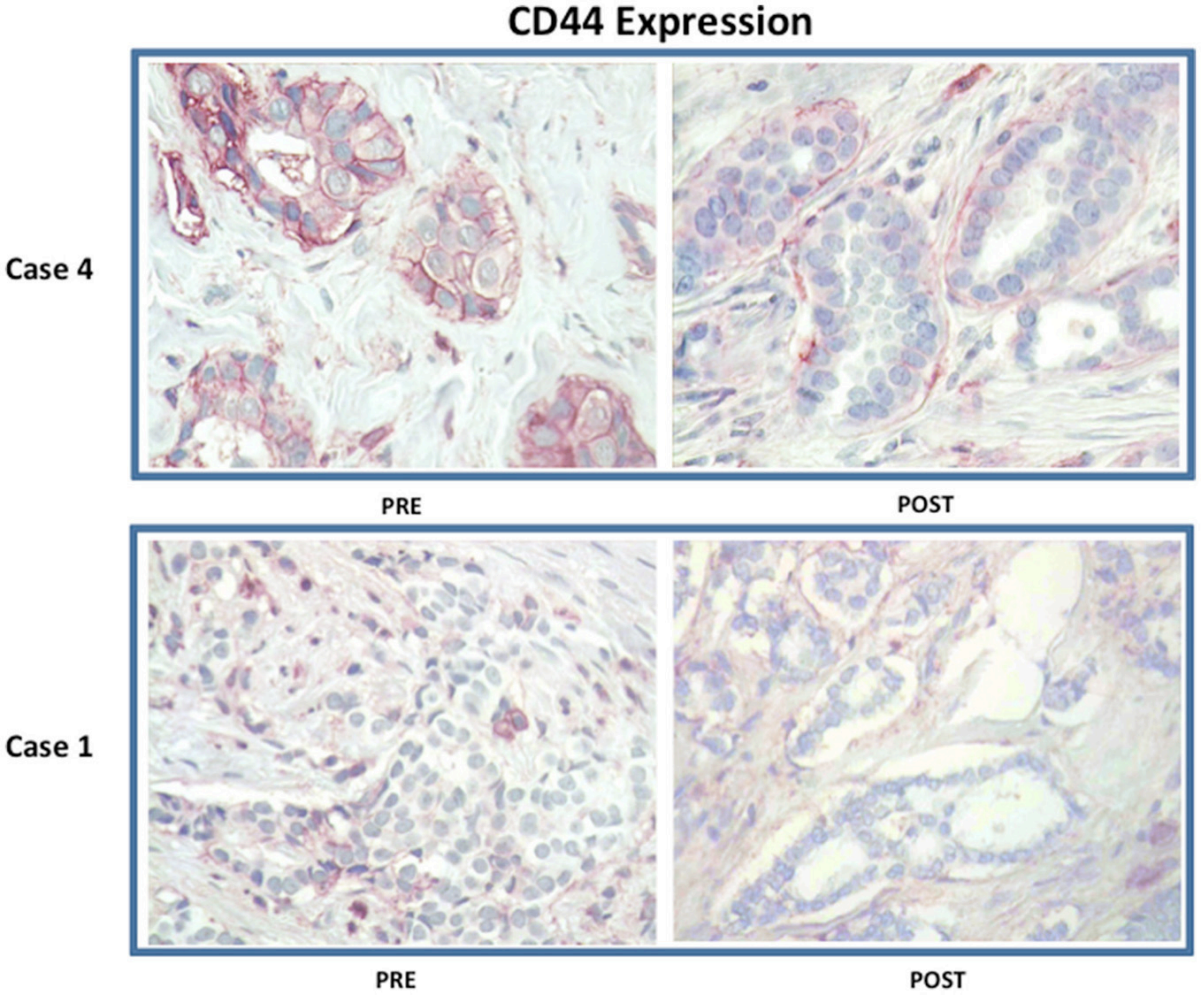
Effect of doxycycline administration on the expression of CD44 in early breast cancer patients: Representative images are shown. Note that treatment with doxycycline reduces the expression of CD44, as seen by immuno-histochemical staining. Representative images from two case are shown. Magnification, 40X.

**Figure 5:**
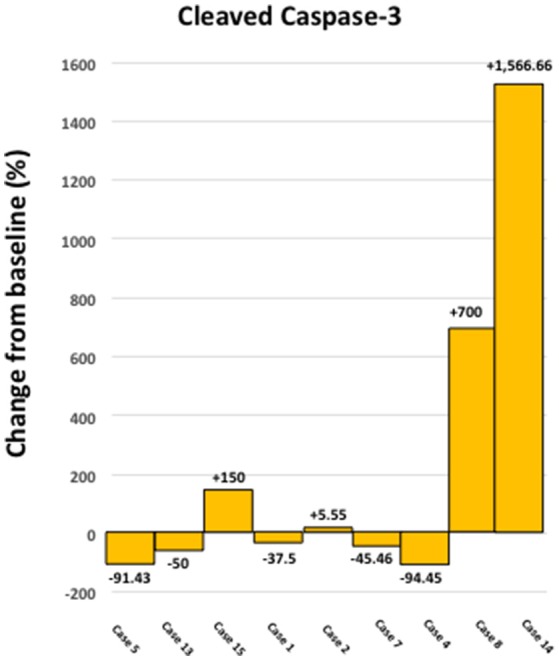
Effect of doxycycline administration on the levels of cleaved caspase 3 in early breast cancer patients. Note that the levels of cleaved caspase 3 showed the largest increases in two patients, which demonstrated the highest reductions in CD44 levels (Cases 8 and14); compare with Figure 3. In addition, the levels of cleaved caspase 3 were increased in 4 out of 9 patients studied (~44 %).

Figure 3 shows a waterfall plot of CD44 expression in the 9 individual breast cancer patients. Remarkably, CD44 levels were reduced between 17.65 and 66.67%, in 8 out of 9 patients treated with doxycycline. Representative images of this reduction in CD44 immuno-staining are illustrated in Figure 4 for two patients. In contrast, only one patient showed a rise in CD44, by 15%. Overall, this represents a positive response rate approaching 90%. It is worth noting that the levels of cleaved caspase-3 were most strikingly elevated in the two patients (Cases 8 & 14) that showed the largest reductions in CD44 expression (Figure 5). Therefore, a certain threshold level may need to be reached to augment the activation of caspase-3.

**Figure 6:**
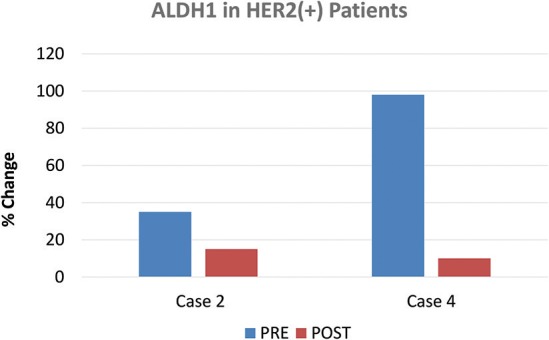
Effect of doxycycline administration on the expression of ALDH1 in HER2(+) early breast cancer patients. The two patients of the HER2(+) sub-type, also showed positivity for another stem cell marker, namely ALDH1. Note that ALDH1 levels were reduced by nearly 60% in one patient (Case 2), while ALDH1 levels were reduced by 90% in the other patient (Case 4), in response to doxycycline.

The two patients of the HER2(+) sub-type, also showed positivity for another stem cell marker, namely ALDH1. Interestingly, ALDH1 levels were reduced by nearly 60% in one patient (Case 2), while ALDH1 levels were reduced by ~90% in the other patient (Case 4) (Figure 6), all in response to doxycycline. These results are also consistent with reductions in CD44; in these same two HER2(+) patients, CD44 levels were reduced by nearly 40% (Case 2) and 60% (Case 4), respectively (Figure 3).

### Status of biomarkers in tumor tissue sections from the untreated control group, before and after surgery

In contrast to our results with the doxycycline treated patient population, patients in the untreated control group did not show any statistically significant changes in the expression of CD44, when tumor tissue sections were compared before and after surgery (Figure S1). The results of multi-variate analysis are included as Supplemental Information (Tables S11–S15) and showed that CD44 remained unchanged (see Table S13; ANOVA; *P* < 0.7707).

Therefore, surgery itself was not sufficient to significantly change the expression levels of the tumor markers examined, including CD44.

## Discussion

Here, we conducted a clinical pilot study with doxycycline, to assess its effects in early breast cancer patients. Importantly, most biomarkers tested remained unchanged, with the exception of CD44, which was reduced on average by nearly 40%, in a period of only two weeks of treatment. Analysis of waterfall plot data revealed that in 8 out of 9 patients treated with doxycycline, CD44 levels were reduced between 17.65 and 66.67%. In contrast, only one patient showed a rise in CD44, by 15%. Two patients of the HER2(+) sub-type, also showed positivity for another stem cell marker, namely ALDH1. In these HER2(+) patients, ALDH1 levels were reduced by nearly 60% in one patient, while ALDH1 levels were reduced by 90% in the other patient, in response to doxycycline. Thus, oral doxycycline treatment effectively reduced the expression of two CSC markers, in early breast cancer patients.

Our current *in vivo* results are consistent with recent findings in MCF7 and MDA-MB-468 cells, two human breast cancer cell lines in culture, which showed significant reductions in the CD44(+)/CD24(-/low) CSC population, after treatment with doxycycline (17). In addition, the expression levels of other “stemness” markers (Oct4, Sox2, Nanog and CD44) were also reduced by >50%, in response to doxycycline, as assessed by mRNA levels and independently confirmed by immuno-blot analysis (17).

Similarly, doxycycline has been shown to reduce ALDH(+) breast CSCs in HER2(+) and triple-negative human breast cancer cell lines *in vitro* (18). As such, doxycycline may be useful for targeting both the CD44(+) and ALDH(+) sub-populations of human breast CSCs (17, 18).

The levels of cleaved caspase-3 appeared to be elevated after treatment with Doxycycline; however, this did not reach statistical significance in all the tumor grades. Nevertheless, Doxycycline has been shown to induce apoptosis in human breast cancer cell lines *in vitro* (17).

## Conclusions

Pre-operative treatment with oral doxycycline (200 mg per day) for 2 weeks is sufficient to reduce both CD44 and ALDH1 expression in tumor tissue from early breast cancer patients. However, additional clinical studies (with larger patient numbers) will be required to further validate these promising clinical pilot studies.

## Materials and methods

### Trial construction, ethical review and EU clinical trial registration

A Phase II clinical trial (pre-operative “window” study; Figure 1) for the use of oral doxycycline in early breast cancer patients was submitted, reviewed, and approved by the local and national ethics committees at the Pisa University Hospital and the Italian Ministry of Health (Rome, Italy). All patients underwent informed written consent, prior to their inclusion in the study. Doxycycline was administered during the “window-of-opportunity,” after diagnosis and exactly 14 days before the date of surgery, while the patient was waiting for tumor excision at surgery. The acronym for the trial is ABC (Antibiotics for Breast Cancer) and the EudraCT registration number is 2016-000871-26. EudraCT is the European Clinical Trials Database (European Union Drug Regulating Authorities Clinical Trials). The full title of the study is: “A Phase II Open-Label Randomized Controlled Pre-Surgical Feasibility Study of Doxycycline in Early Breast Cancer.” The objective and primary goal of the trial is: To determine whether short-term (2-weeks) pre-operative treatment with oral doxycycline of stage I-to-III early breast cancer patients results in inhibition of tumor proliferation markers, as determined by a reduction in tumor Ki67 from baseline (pre-treatment) to post-treatment (at time of surgical excision). Doxycycline (Bassado-brand) was administered orally, 100-mg twice a day for a total of 200-mg per day, for a period of 14-days. During this period, the control group received no medical therapy (i.e., standard of care: waiting for surgery). Information about study subjects is kept confidential and managed according to the requirements of the EU and Italian regulations. All of our breast cancer cases were NST (No Special Type, invasive carcinomas), previously known as “ductal” carcinomas.

### Plasma doxycycline levels

Doxycycline oral intake was validated by measuring the concentrations in plasma samples, obtained immediately prior to surgery [mean +/– SD, 0.76 ± 0.41 mg/L, range 0.25–1.57 mg/L]. Doxycycline levels were determined by mass spectrometry analysis. This precise monitoring confirmed the compliance of patients to the planned treatment regimen, proposed to them at the time of enrollment.

### Immuno-staining reagents

Antibodies for immuno-staining were purchased from commercial sources, as briefly summarized in Table S16.

### Immuno-staining and quantitation

Tumor expression of Ki67, p27, cleaved caspase 3, CD31, CSC markers (CD44, ALDH1), and mitochondria (TOMM20) was performed on formalin-fixed paraffin-embedded tumor tissues. Tissue sections (4 micron) were de-paraffinized with xylene and rehydrated through a graded alcohol series. After rinsing with phosphate buffer saline (PBS) sample were immersed in sodium citrate buffer (pH 6) for p27 and cleaved caspase 3 and in UNMASKER buffer (pH 7,8) for CD44, TOMM20, ADLH1, and heated in a microwave oven at 100°C. The endogenous peroxidase was blocked by 10 min incubation in 3% H_2_O_2_. After blocking with normal goat serum for 10 min at room temperature, the slides were further incubated overnight at 4°C with the following primary antibodies: mouse anti CD44 (1:1000, clone 156-3C11), mouse anti-ALDH1A1, (1:500, clone 703410), rabbit anti cleaved caspase 3 (Asp175; 1:150), rabbit anti-p27 (1:250) and mouse anti TOMM20 (1:250, clone F-10). A biotin conjugated goat derived secondary antibody was applied followed by the enzyme-labeled streptavidin and substrate chromogen (Rabbit/Mouse specific HRP/DAB-ABC detection IHC kit, Abcam). Slides were counterstained with hematoxylin.

The immunostaining for Ki67 (ready to use, clone MIB-1, Dako) and CD31 (ready to use, clone JC70, Ventana Medical Systems) instead was performed in an automated immunostainer (BenchMark Ultra, Ventana Medical Systems). Staining intensity and percentage of positive tumor cells was measured. Ki67 is a nuclear marker expressed in all phases of the cell cycle except G_0_. The “Ki67 index” (percentage of nuclei showing nuclear immuno-reactivity of any intensity) was determined as per routine protocols. p27 (nuclear staining) is a cell cycle inhibitor that negatively correlates with Ki67. Caspase-3 (cytoplasmic and/or nuclear staining) is synthesized as an inactive pro-enzyme which is activated by cleavage in cells undergoing apoptosis. CD31 (membranous staining) is expressed by endothelial cells and is used as a marker of angiogenesis. CD44 (membranous staining: complete or incomplete, of any intensity) and ALDH1 (cytoplasmic staining) are well-established markers that are elevated in cells with “stem-like” characteristics. TOMM20 (cytoplasmic staining), a central component of the receptor complex responsible for the recognition and translocation of cytosolically synthesized mitochondrial preproteins, is used as a marker of mitochondria. Staining percentage of positive tumor cells was measured independently by two blinded-pathologists. Discrepancies in interpretation or scoring (< 5% of cases) were resolved by consensus conference at a double-headed microscope. All changes in tumor markers were analyzed as a percentage (pre-post/pre x 100) and an absolute (pre-post) change from baseline.

### Statistical analysis

The values of the markers before the treatment were our reference (100%), and all the other values measured after the treatment are presented as a post/pre ratio, to point out any increases or decreases from the reference value. The values in the graphs are represented by the average value of each endpoint, with relative standard error of the mean (SEM). The significant differences were assessed with MedCalc 12 (unpaired *t-*test). Values of *p* < 0.05 were considered statistically significant. Multi-variate analysis with ANOVA was also carried out and the results of this analysis are included as Supplementary Information (see Tables S1–S15).

## Author contributions

The ABC trial is being carried out at the Breast Care Center at the Pisa University Hospital. Patient recruitment is led by the surgeons (MR and MG). FS and ML initially conceived the idea of a doxycycline-based breast cancer clinical trial and wrote a first draft of the clinical trial. CS, MR, AD, GF, CM, MG, CMM, and AN edited and implemented the clinical trial. CS processed the tissue samples and generated the final figures. MM performed immuno-staining on the tissue sections. AD performed the analysis of the doxycycline blood dosages. PA performed the statistical analyses. FS and ML wrote the first draft of the paper, which was edited and approved by all the co-authors.

### Conflict of interest statement

The authors declare that the research was conducted in the absence of any commercial or financial relationships that could be construed as a potential conflict of interest.

## Supplementary material

Figure S1 Expression of six different classes of biomarkers in early breast cancer patients. In contrast to our results with the doxycycline treated patient population, patients in the untreated control group did not show any significant changes in the expression of tumor markers, when tumor sections were compared, before and after surgery.
