# Supplementary material for: Doxycycline, an Inhibitor of Mitochondrial Biogenesis, Effectively Reduces Cancer Stem Cells (CSCs) in Early Breast Cancer Patients: A Clinical Pilot Study
Source: Front Oncol. 2018 Oct 12;8:452. doi: 10.3389/fonc.2018.00452 (PMC6194352; doi:10.3389/fonc.2018.00452)
Supplement: Supplementary file 1 [file Table_1.DOCX]

**Supplementary Information: DOXY-TREATED PATIENTS (Tables S1 to S10).**

**Table S1-A: ANOVA for KI67 - Type III Sums of Squares**

| *Source* | *Sum of Squares* | *Df* | *Mean Square* | *F-Ratio* | *P-Value* |
| --- | --- | --- | --- | --- | --- |
| MAIN EFFECTS |  |  |  |  |  |
| A: Time (Pre/Post) | 66.24 | 1 | 66.24 | 0.21 | 0.6516 |
| B: Histological Grade | 1103.65 | 2 | 551.826 | 1.78 | 0.2074 |
| C: Diameter Type | 312.097 | 1 | 312.097 | 1.01 | 0.3340 |
| RESIDUAL | 4030.5 | 13 | 310.038 |  |  |
| TOTAL (CORRECTED) | 5337.75 | 17 |  |  |  |

All F-ratios are based on the residual mean square error. Since no P-values are less than 0.05, none of

the factors have a statistically significant effect on KI67 at the 95.0% confidence level.

**Table S1-B: Least Squares Means for KI67 with 95.0 Percent Confidence Intervals**

|  |  |  | *Stnd.* | *Lower* | *Upper* |
| --- | --- | --- | --- | --- | --- |
| *Level* | *Count* | *Mean* | *Error* | *Limit* | *Limit* |
| GRAND MEAN | 18 | 94.0367 |  |  |  |
| Time |  |  |  |  |  |
| POST | 9 | 95.9551 | 7.07864 | 80.6626 | 111.248 |
| PRE | 9 | 92.1184 | 7.07864 | 76.8259 | 107.411 |
| HISTOLOGICAL GRADE |  |  |  |  |  |
| G1 | 2 | 78.717 | 13.2725 | 50.0436 | 107.391 |
| G2 | 12 | 104.481 | 5.30898 | 93.0113 | 115.95 |
| G3 | 4 | 98.9125 | 8.80395 | 79.8927 | 117.932 |
| DIAMETER TYPE |  |  |  |  |  |
| Large | 6 | 89.4238 | 8.53299 | 70.9893 | 107.858 |
| Small | 12 | 98.6497 | 5.93562 | 85.8265 | 111.473 |

This table shows the mean KI67 for each level of the factors. It also shows the standard error of each mean,

which is a measure of its sampling variability. The rightmost two columns show 95.0% confidence intervals for

each of the means.

**Table S2-A: ANOVA for TOMM20 - Type III Sums of Squares**

| *Source* | *Sum of Squares* | *Df* | *Mean Square* | *F-Ratio* | *P-Value* |
| --- | --- | --- | --- | --- | --- |
| MAIN EFFECTS |  |  |  |  |  |
| A: Time (Pre/Post) | 2.66036 | 1 | 2.66036 | 0.01 | 0.9060 |
| B: Histological Grade | 769.049 | 2 | 384.525 | 2.10 | 0.1626 |
| C: Diameter Type | 61.2137 | 1 | 61.2137 | 0.33 | 0.5734 |
| RESIDUAL | 2385.09 | 13 | 183.469 |  |  |
| TOTAL (CORRECTED) | 3156.82 | 17 |  |  |  |

All F-ratios are based on the residual mean square error. Since no P-values are less than 0.05, none of

the factors have a statistically significant effect on TOMM20 at the 95.0% confidence level.

**Table S2-B: Least Squares Means for TOMM20 with 95.0 Percent Confidence Intervals**

|  |  |  | *Stnd.* | *Lower* | *Upper* |
| --- | --- | --- | --- | --- | --- |
| *Level* | *Count* | *Mean* | *Error* | *Limit* | *Limit* |
| GRAND MEAN | 18 | 104.347 |  |  |  |
| Time |  |  |  |  |  |
| POST | 9 | 104.731 | 5.44532 | 92.9671 | 116.495 |
| PRE | 9 | 103.962 | 5.44532 | 92.1982 | 115.726 |
| HISTOLOGICAL GRADE |  |  |  |  |  |
| G1 | 2 | 118.708 | 10.21 | 96.6506 | 140.765 |
| G2 | 12 | 100.554 | 4.08399 | 91.7314 | 109.377 |
| G3 | 4 | 93.7775 | 6.77253 | 79.1463 | 108.409 |
| DIAMETER TYPE |  |  |  |  |  |
| Large | 6 | 106.39 | 6.5641 | 92.2086 | 120.57 |
| Small | 12 | 102.304 | 4.56604 | 92.4393 | 112.168 |

This table shows the mean TOMM20 for each level of the factors. It also shows the standard error of each mean,

which is a measure of its sampling variability. The rightmost two columns show 95.0% confidence intervals for

each of the means.

**Table S3-A: ANOVA for CD44 - Type III Sums of Squares**

| *Source* | *Sum of Squares* | *Df* | *Mean Square* | *F-Ratio* | *P-Value* |
| --- | --- | --- | --- | --- | --- |
| MAIN EFFECTS |  |  |  |  |  |
| A: Time (Pre/Post) | 6073.49 | 1 | 6073.49 | 19.51 | **0.0007** |
| B: Histological Grade | 970.107 | 2 | 485.053 | 1.56 | 0.2474 |
| C: Diameter Type | 439.533 | 1 | 439.533 | 1.41 | 0.2560 |
| RESIDUAL | 4047.04 | 13 | 311.311 |  |  |
| TOTAL (CORRECTED) | 11380.6 | 17 |  |  |  |

All F-ratios are based on the residual mean square error. Since one P-value is less than 0.05, this factor has

a statistically significant effect on CD44 at the 95.0% confidence level.

**Table S3-B: Least Squares Means for CD44 with 95.0 Percent Confidence Intervals**

|  |  |  | *Stnd.* | *Lower* | *Upper* |
| --- | --- | --- | --- | --- | --- |
| *Level* | *Count* | *Mean* | *Error* | *Limit* | *Limit* |
| GRAND MEAN | 18 | 73.7445 |  |  |  |
| Time |  |  |  |  |  |
| POST | 9 | 55.3756 | 7.09315 | 40.0517 | 70.6994 |
| PRE | 9 | 92.1134 | 7.09315 | 76.7895 | 107.437 |
| HISTOLOGICAL GRADE |  |  |  |  |  |
| G1 | 2 | 61.1907 | 13.2997 | 32.4584 | 89.9229 |
| G2 | 12 | 84.2452 | 5.31987 | 72.7523 | 95.7381 |
| G3 | 4 | 75.7975 | 8.822 | 56.7387 | 94.8563 |
| DIAMETER TYPE |  |  |  |  |  |
| Large | 6 | 68.2702 | 8.55049 | 49.7979 | 86.7424 |
| Small | 12 | 79.2188 | 5.94779 | 66.3693 | 92.0682 |

This table shows the mean CD44 for each level of the factors. It also shows the standard error of each mean,

which is a measure of its sampling variability. The rightmost two columns show 95.0% confidence intervals for

each of the means..

**Table S4-A: ANOVA for CLEAVED CASPASE-3 - Type III Sums of Squares**

| *Source* | *Sum of Squares* | *Df* | *Mean Square* | *F-Ratio* | *P-Value* |
| --- | --- | --- | --- | --- | --- |
| MAIN EFFECTS |  |  |  |  |  |
| A: Time (Pre/Post) | 245787. | 1 | 245787.0 | 2.30 | 0.1530 |
| B: Histological Grade | 1.11068E6 | 2 | 555342.0 | 5.20 | **0.0219** |
| C: Diameter Type | 95407.9 | 1 | 95407.9 | 0.89 | 0.3616 |
| RESIDUAL | 1.38721E6 | 13 | 106708.0 |  |  |
| TOTAL (CORRECTED) | 2.74442E6 | 17 |  |  |  |

All F-ratios are based on the residual mean square error. Since one P-value is less than 0.05, this factor has

a statistically significant effect on CLEAVED CASPASE-3 at the 95.0% confidence level.

**Table S4-B: Least Squares Means for CLEAVED CASPASE-3 with 95.0 Percent Confidence Intervals**

|  |  |  | *Stnd.* | *Lower* | *Upper* |
| --- | --- | --- | --- | --- | --- |
| *Level* | *Count* | *Mean* | *Error* | *Limit* | *Limit* |
| GRAND MEAN | 18 | 406.926 |  |  |  |
| Time |  |  |  |  |  |
| POST | 9 | 523.78 | 131.323 | 240.073 | 807.487 |
| PRE | 9 | 290.072 | 131.323 | 6.36476 | 573.779 |
| HISTOLOGICAL GRADE |  |  |  |  |  |
| G1 | 2 | 963.984 | 246.231 | 432.033 | 1495.94 |
| G2 | 12 | 179.019 | 98.4925 | -33.7616 | 391.799 |
| G3 | 4 | 77.775 | 163.331 | -275.082 | 430.632 |
| DIAMETER TYPE |  |  |  |  |  |
| Large | 6 | 487.58 | 158.304 | 145.583 | 829.577 |
| Small | 12 | 326.272 | 110.118 | 88.3761 | 564.168 |

This table shows the mean CLEAVED CASPASE-3 for each level of the factors. It also shows the standard error of each mean,

which is a measure of its sampling variability. The rightmost two columns show 95.0% confidence intervals for

each of the means.

**Table S5-A.** **ANOVA for p27 - Type III Sums of Squares**

| *Source* | *Sum of Squares* | *Df* | *Mean Square* | *F-Ratio* | *P-Value* |
| --- | --- | --- | --- | --- | --- |
| MAIN EFFECTS |  |  |  |  |  |
| A: Time (Pre/Post) | 1102.93 | 1 | 1102.93 | 0.11 | 0.7482 |
| B: Histological Grade | 38901.8 | 2 | 19450.9 | 1.90 | 0.1895 |
| C: Diameter Type | 17391.3 | 1 | 17391.3 | 1.69 | 0.2156 |
| RESIDUAL | 133398. | 13 | 10261.4 |  |  |
| TOTAL (CORRECTED) | 182993. | 17 |  |  |  |

All F-ratios are based on the residual mean square error. Since no P-values are less than 0.05, none of

the factors have a statistically significant effect on p27 at the 95.0% confidence level.

**Table S5-B. Least Squares Means for p27 with 95.0 Percent Confidence Intervals**

|  |  |  | *Stnd.* | *Lower* | *Upper* |
| --- | --- | --- | --- | --- | --- |
| *Level* | *Count* | *Mean* | *Error* | *Limit* | *Limit* |
| GRAND MEAN | 18 | 107.498 |  |  |  |
| Time |  |  |  |  |  |
| POST | 9 | 115.326 | 40.7235 | 27.3481 | 203.304 |
| PRE | 9 | 99.6706 | 40.7235 | 11.6925 | 187.649 |
| HISTOLOGICAL GRADE |  |  |  |  |  |
| G1 | 2 | 65.565 | 76.3566 | -99.3938 | 230.524 |
| G2 | 12 | 71.93 | 30.5426 | 5.94648 | 137.914 |
| G3 | 4 | 185.0 | 50.6492 | 75.5787 | 294.421 |
| DIAMETER TYPE |  |  |  |  |  |
| Large | 6 | 73.0633 | 49.0904 | -32.9903 | 179.117 |
| Small | 12 | 141.933 | 34.1477 | 68.1615 | 215.705 |

This table shows the mean p27 for each level of the factors. It also shows the standard error of each mean,

which is a measure of its sampling variability. The rightmost two columns show 95.0% confidence intervals for

each of the means.

**Table S6-A: ANOVA for KI67 - Type III Sums of Squares**

| *Source* | *Sum of Squares* | *Df* | *Mean Square* | *F-Ratio* | *P-Value* |
| --- | --- | --- | --- | --- | --- |
| MAIN EFFECTS |  |  |  |  |  |
| A: Time (Pre/Post) | 66.2401 | 1 | 66.2401 | 0.19 | 0.6690 |
| B: Molecular Subtype | 46.4659 | 1 | 46.4659 | 0.13 | 0.7200 |
| RESIDUAL | 5225.04 | 15 | 348.336 |  |  |
| TOTAL (CORRECTED) | 5337.75 | 17 |  |  |  |

All F-ratios are based on the residual mean square error. Since no P-values are less than 0.05, none of

the factors have a statistically significant effect on KI67 at the 95.0% confidence level.

**Table S6-B: Table of Least Squares Means for KI67 with 95.0 Percent Confidence Intervals**

|  |  |  | *Stnd.* | *Lower* | *Upper* |
| --- | --- | --- | --- | --- | --- |
| *Level* | *Count* | *Mean* | *Error* | *Limit* | *Limit* |
| GRAND MEAN | 18 | 100.845 |  |  |  |
| Time |  |  |  |  |  |
| POST | 9 | 102.763 | 6.88065 | 88.0974 | 117.429 |
| PRE | 9 | 98.9265 | 6.88065 | 84.2607 | 113.592 |
| MOLECULAR SUBTYPE |  |  |  |  |  |
| HER2(+) | 4 | 98.9125 | 9.33188 | 79.022 | 118.803 |
| Luminal | 14 | 102.777 | 4.9881 | 92.1452 | 113.409 |

This table shows the mean KI67 for each level of the factors. It also shows the standard error of each mean,

which is a measure of its sampling variability. The rightmost two columns show 95.0% confidence intervals for

each of the means.

**Table S7-A: ANOVA for TOMM20 - Type III Sums of Squares**

| *Source* | *Sum of Squares* | *Df* | *Mean Square* | *F-Ratio* | *P-Value* |
| --- | --- | --- | --- | --- | --- |
| MAIN EFFECTS |  |  |  |  |  |
| A: Time (Pre/Post) | 2.66036 | 1 | 2.66036 | 0.01 | 0.9086 |
| B: Molecular Subtype | 224.495 | 1 | 224.495 | 1.15 | 0.3006 |
| RESIDUAL | 2929.67 | 15 | 195.311 |  |  |
| TOTAL (CORRECTED) | 3156.82 | 17 |  |  |  |

All F-ratios are based on the residual mean square error. Since no P-values are less than 0.05, none of

the factors have a statistically significant effect on TOMM20 at the 95.0% confidence level.

**Table S7-B: Least Squares Means for TOMM20 with 95.0 Percent Confidence Intervals**

|  |  |  | *Stnd.* | *Lower* | *Upper* |
| --- | --- | --- | --- | --- | --- |
| *Level* | *Count* | *Mean* | *Error* | *Limit* | *Limit* |
| GRAND MEAN | 18 | 98.0248 |  |  |  |
| Time |  |  |  |  |  |
| POST | 9 | 98.4093 | 5.15221 | 87.4276 | 109.391 |
| PRE | 9 | 97.6404 | 5.15221 | 86.6587 | 108.622 |
| MOLECULAR SUBTYPE |  |  |  |  |  |
| HER2(+) | 4 | 93.7775 | 6.98769 | 78.8836 | 108.671 |
| Luminal | 14 | 102.272 | 3.73508 | 94.311 | 110.233 |

This table shows the mean TOMM20 for each level of the factors. It also shows the standard error of each mean,

which is a measure of its sampling variability. The rightmost two columns show 95.0% confidence intervals for

each of the means.

**Table S8-A: ANOVA for CD44 - Type III Sums of Squares**

| *Source* | *Sum of Squares* | *Df* | *Mean Square* | *F-Ratio* | *P-Value* |
| --- | --- | --- | --- | --- | --- |
| MAIN EFFECTS |  |  |  |  |  |
| A: Time (Pre/Post) | 6073.49 | 1 | 6073.49 | 17.75 | **0.0008** |
| B: Molecular Subtype | 175.017 | 1 | 175.017 | 0.51 | 0.4855 |
| RESIDUAL | 5132.09 | 15 | 342.14 |  |  |
| TOTAL (CORRECTED) | 11380.6 | 17 |  |  |  |

All F-ratios are based on the residual mean square error. Since one P-value is less than 0.05, this factor has

a statistically significant effect on CD44 at the 95.0% confidence level.

**Table S8-B: Table of Least Squares Means for CD44 with 95.0 Percent Confidence Intervals**

|  |  |  | *Stnd.* | *Lower* | *Upper* |
| --- | --- | --- | --- | --- | --- |
| *Level* | *Count* | *Mean* | *Error* | *Limit* | *Limit* |
| GRAND MEAN | 18 | 79.5477 |  |  |  |
| Time |  |  |  |  |  |
| POST | 9 | 61.1788 | 6.81917 | 46.644 | 75.7135 |
| PRE | 9 | 97.9166 | 6.81917 | 83.3818 | 112.451 |
| MOLECULAR SUBTYPE |  |  |  |  |  |
| HER2(+) | 4 | 75.7975 | 9.24851 | 56.0847 | 95.5103 |
| Luminal | 14 | 83.2979 | 4.94354 | 72.7609 | 93.8348 |

This table shows the mean CD44 for each level of the factors. It also shows the standard error of each mean,

which is a measure of its sampling variability. The rightmost two columns show 95.0% confidence intervals

for each of the means.

**Table S9-A: ANOVA for CLEAVED CASPASE-3 - Type III Sums of Squares**

| *Source* | *Sum of Squares* | *Df* | *Mean Square* | *F-Ratio* | *P-Value* |
| --- | --- | --- | --- | --- | --- |
| MAIN EFFECTS |  |  |  |  |  |
| A: Time (Pre/Post) | 245787.0 | 1 | 245787. | 1.54 | 0.2342 |
| B: Molecular Subtype | 99478.0 | 1 | 99478.0 | 0.62 | 0.4426 |
| RESIDUAL | 2.39916E6 | 15 | 159944. |  |  |
| TOTAL (CORRECTED) | 2.74442E6 | 17 |  |  |  |

All F-ratios are based on the residual mean square error. Since no P-values are less than 0.05, none of

the factors have a statistically significant effect on CLEAVED CASPASE-3 at the 95.0% confidence level.

**Table S9-B: Least Squares Means for CLEAVED CASPASE-3 with 95.0 Percent Confidence Intervals**

|  |  |  | *Stnd.* | *Lower* | *Upper* |
| --- | --- | --- | --- | --- | --- |
| *Level* | *Count* | *Mean* | *Error* | *Limit* | *Limit* |
| GRAND MEAN | 18 | 167.183 |  |  |  |
| Time |  |  |  |  |  |
| POST | 9 | 284.037 | 147.439 | -30.2236 | 598.297 |
| PRE | 9 | 50.329 | 147.439 | -263.931 | 364.589 |
| MOLECULAR SUBTYPE |  |  |  |  |  |
| HER2(+) | 4 | 77.775 | 199.965 | -348.441 | 503.991 |
| Luminal | 14 | 256.591 | 106.886 | 28.7688 | 484.413 |

This table shows the mean CLEAVED CASPASE-3 for each level of the factors. It also shows the standard error of each mean,

which is a measure of its sampling variability. The rightmost two columns show 95.0% confidence intervals for

each of the means.

**Table S10-A. ANOVA for p27 - Type III Sums of Squares**

| *Source* | *Sum of Squares* | *Df* | *Mean Square* | *F-Ratio* | *P-Value* |
| --- | --- | --- | --- | --- | --- |
| MAIN EFFECTS |  |  |  |  |  |
| A: Time (Pre/Post) | 1102.93 | 1 | 1102.93 | 0.11 | 0.7454 |
| B: Molecular Subtype | 30628.6 | 1 | 30628.6 | 3.04 | 0.1018 |
| RESIDUAL | 151261.0 | 15 | 10084.1 |  |  |
| TOTAL (CORRECTED) | 182993.0 | 17 |  |  |  |

All F-ratios are based on the residual mean square error. Since no P-values are less than 0.05, none of

the factors have a statistically significant effect on p27 at the 95.0% confidence level.

**Table S10-B. Least Squares Means for p27 with 95.0 Percent Confidence Intervals**

|  |  |  | *Stnd.* | *Lower* | *Upper* |
| --- | --- | --- | --- | --- | --- |
| *Level* | *Count* | *Mean* | *Error* | *Limit* | *Limit* |
| GRAND MEAN | 18 | 135.389 |  |  |  |
| Time |  |  |  |  |  |
| POST | 9 | 143.217 | 37.021 | 64.3085 | 222.126 |
| PRE | 9 | 127.562 | 37.021 | 48.6529 | 206.47 |
| MOLECULAR SUBTYPE |  |  |  |  |  |
| HER2(+) | 4 | 185.0 | 50.2098 | 77.9802 | 292.02 |
| Luminal | 14 | 85.7786 | 26.8382 | 28.5741 | 142.983 |

This table shows the mean p27 for each level of the factors. It also shows the standard error of each mean,

which is a measure of its sampling variability. The rightmost two columns show 95.0% confidence intervals for

each of the means.
