# Supplementary material for: Doxycycline, an Inhibitor of Mitochondrial Biogenesis, Effectively Reduces Cancer Stem Cells (CSCs) in Early Breast Cancer Patients: A Clinical Pilot Study
Source: Front Oncol. 2018 Oct 12;8:452. doi: 10.3389/fonc.2018.00452 (PMC6194352; doi:10.3389/fonc.2018.00452)
Supplement: Supplementary file 2 [file Table_2.DOCX]

**Supplementary Information: UN-TREATED PATIENTS (Tables S11 to S15).**

**Table S11-A: ANOVA for Ki67 - Type III Sums of Squares**

| *Source* | *Sum of Squares* | *Df* | *Mean Square* | *F-Ratio* | *P-Value* |
| --- | --- | --- | --- | --- | --- |
| MAIN EFFECTS |  |  |  |  |  |
| A: Time (Pre/Post) | 2559.09 | 1 | 2559.09 | 3.60 | 0.0995 |
| B: Hisological Grade | 675.6 | 1 | 675.6 | 0.95 | 0.3619 |
| C: Diameter Type | 187.388 | 1 | 187.388 | 0.26 | 0.6233 |
| D: Molecular Subtype | 489.426 | 1 | 489.426 | 0.69 | 0.4339 |
| RESIDUAL | 4971.79. 95 | 7 | 710.25 |  |  |
| TOTAL (CORRECTED) | 8300.17 | 11 |  |  |  |

All F-ratios are based on the residual mean square error. Since no P-values are less than 0.05, none of

the factors have a statistically significant effect on Ki67 at the 95.0% confidence level.

**Table S11-B: Least Squares Means for Ki67 with 95.0 Percent Confidence Intervals**

|  |  |  | *Stnd.* | *Lower* | *Upper* |
| --- | --- | --- | --- | --- | --- |
| *Level* | *Count* | *Mean* | *Error* | *Limit* | *Limit* |
| GRAND MEAN | 12 | 123.635 |  |  |  |
| Time |  |  |  |  |  |
| POST | 6 | 138.238 | 15.3867 | 101.855 | 174.622 |
| PRE | 6 | 109.032 | 15.3867 | 72.6479 | 145.415 |
| HISTOLOGICAL GRADE |  |  |  |  |  |
| G2 | 6 | 134.89 | 22.0974 | 82.6377 | 187.142 |
| G3 | 6 | 112.38 | 11.54 | 85.0921 | 139.668 |
| DIAMETER TYPE |  |  |  |  |  |
| Large | 6 | 129.563 | 22.0974 | 77.3102 | 181.815 |
| Small | 6 | 117.708 | 11.54 | 90.4196 | 144.995 |
| MOLECULAR SUBTYPE |  |  |  |  |  |
| Luminal | 10 | 110.087 | 9.42238 | 87.8071 | 132.368 |
| Luminal/HER2(+) | 2 | 137.182 | 28.2671 | 70.3412 | 204.024 |

This table shows the mean Ki67 for each level of the factors. It also shows the standard error of each mean,

which is a measure of its sampling variability. The rightmost two columns show 95.0% confidence intervals

for each of the means.

**Table S12: ANOVA for TOMM20 - Type III Sums of Squares**

| *Source* | *Sum of Squares* | *Df* | *Mean Square* | *F-Ratio* | *P-Value* |
| --- | --- | --- | --- | --- | --- |
| MAIN EFFECTS |  |  |  |  |  |
| A: Time (Pre/Post) | 25.6961 | 1 | 25.6961 | 1.10 | 0.3282 |
| B: Histological Grade | 43.8154 | 1 | 43.8154 | 1.88 | 0.2123 |
| C: Diameter Type | 10.5656 | 1 | 10.5656 | 0.45 | 0.5220 |
| D: Molecular Subtype | 6.4792 | 1 | 6.4792 | 0.28 | 0.6140 |
| RESIDUAL | 162.837 | 7 | 23.2624 |  |  |
| TOTAL (CORRECTED) | 348.108 | 11 |  |  |  |

All F-ratios are based on the residual mean square error. Since no P-values are less than 0.05, none of

the factors have a statistically significant effect on TOMM20 at the 95.0% confidence level.

**Table S13: ANOVA for CD44 - Type III Sums of Squares**

| *Source* | *Sum of Squares* | *Df* | *Mean Square* | *F-Ratio* | *P-Value* |
| --- | --- | --- | --- | --- | --- |
| MAIN EFFECTS |  |  |  |  |  |
| A: Time (Pre/Post) | 328.339 | 1 | 328.339 | 0.09 | 0.7707 |
| B: Histological Grade | 10452.4 | 1 | 10452.4 | 2.92 | 0.1311 |
| C: Diameter Type | 2897.28 | 1 | 2897.28 | 0.81 | 0.3981 |
| D: Molecular Subtype | 5744.94 | 1 | 5744.94 | 1.61 | 0.2456 |
| RESIDUAL | 25042.6 | 7 | 3577.52 |  |  |
| TOTAL (CORRECTED) | 36422.7 | 11 |  |  |  |

All F-ratios are based on the residual mean square error. Since no P-values are less than 0.05, none of

the factors have a statistically significant effect on CD44 at the 95.0% confidence level.

**Table S14: ANOVA for CLEAVED CASPASE-3 - Type III Sums of Squares**

| *Source* | *Sum of Squares* | *Df* | *Mean Square* | *F-Ratio* | *P-Value* |
| --- | --- | --- | --- | --- | --- |
| MAIN EFFECTS |  |  |  |  |  |
| A: Time (Pre/Post) | 3755.94 | 1 | 3755.94 | 4.42 | 0.0736 |
| B: Histological Grade | 370.741 | 1 | 370.741 | 0.44 | 0.5301 |
| C: Diameter Type | 257.613 | 1 | 257.613 | 0.30 | 0.5991 |
| D: Molecular Subtype | 138.72 | 1 | 138.72 | 0.16 | 0.6983 |
| RESIDUAL | 5949.25 | 7 | 849.892 |  |  |
| TOTAL (CORRECTED) | 11884.3 | 11 |  |  |  |

All F-ratios are based on the residual mean square error. Since no P-values are less than 0.05, none of

the factors have a statistically significant effect on CLEAVED CASPASE-3 at the 95.0% confidence level.

**Table S15. ANOVA for p27 - Type III Sums of Squares**

| *Source* | *Sum of Squares* | *Df* | *Mean Square* | *F-Ratio* | *P-Value* |
| --- | --- | --- | --- | --- | --- |
| MAIN EFFECTS |  |  |  |  |  |
| A: Time (Pre/Post) | 426.021 | 1 | 426.021 | 2.19 | 0.1827 |
| B: Histological Grade | 17.0408 | 1 | 17.0408 | 0.09 | 0.7760 |
| C: Diameter Type | 272.653 | 1 | 272.653 | 1.40 | 0.2753 |
| D: Molecular Subtype | 8.52042 | 1 | 8.52042 | 0.04 | 0.8403 |
| RESIDUAL | 1363.27 | 7 | 194.752 |  |  |
| TOTAL (CORRECTED) | 2232.35 | 11 |  |  |  |

All F-ratios are based on the residual mean square error. Since no P-values are less than 0.05, none of

the factors have a statistically significant effect on p27 at the 95.0% confidence level.
