# Supplementary material for: Doxycycline, an Inhibitor of Mitochondrial Biogenesis, Effectively Reduces Cancer Stem Cells (CSCs) in Early Breast Cancer Patients: A Clinical Pilot Study
Source: Front Oncol. 2018 Oct 12;8:452. doi: 10.3389/fonc.2018.00452 (PMC6194352; doi:10.3389/fonc.2018.00452)
Supplement: Supplementary file 3 [file Table_3.DOCX]

**Supplemental Table S16**

**Antibodies Used for Immuno-staining and their Sources.**

**________________________________________________________________________________________**

**Antibody Commercial Source Catalogue Number**

_________________________________________________________________

1. Ki67 Dako M7240

2. p27 Novus Biologicals NBP1-32213

3. Cleaved Caspase-3 Cell Signaling Technology 9661

4. CD31 Ventana Medical Systems 760-4378

5. CD44 ThermoFisher Scientific MA5-13890

6. ALDH1 Novus Biologicals MAB5869

7. TOMM20 Santa Cruz Biotechnology sc-17764

**_________________________________________________________________________________________**
