# Supplementary figures and images for: Doxycycline, an Inhibitor of Mitochondrial Biogenesis, Effectively Reduces Cancer Stem Cells (CSCs) in Early Breast Cancer Patients: A Clinical Pilot Study
Source: Front Oncol. 2018 Oct 12;8:452. doi: 10.3389/fonc.2018.00452 (PMC6194352; doi:10.3389/fonc.2018.00452)

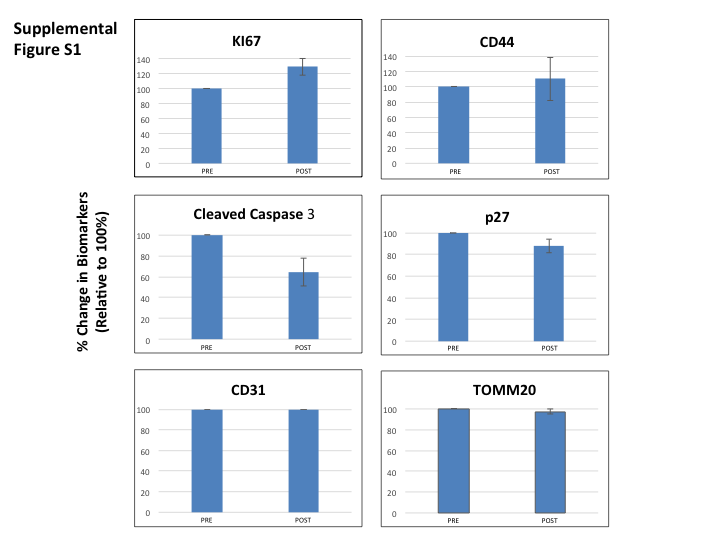

Supplement: Figure S1 — Expression of six different classes of biomarkers in early breast cancer patients. In contrast to our results with the doxycycline treated patient population, patients in the untreated control group did not show any significant changes in the expression of tumor markers, when tumor sections were compared, before and after surgery. [file Image_1.tiff]
